# Retrospective Evaluation of Septic Subtendinous Calcaneal Bursitis in 29 Cattle

**DOI:** 10.3390/ani11051446

**Published:** 2021-05-18

**Authors:** Johann Kofler, Florian Sullmann

**Affiliations:** University Clinic for Ruminants, Department of Farm Animals and Veterinary Public Health, University of Veterinary Medicine Vienna, 1210 Vienna, Austria; floriansullmann@yahoo.de

**Keywords:** hock lesions, septic bursitis, subtendinous calcaneal bursa, surgical debridement, postoperative survival time, cattle

## Abstract

**Simple Summary:**

Infections of the subtendinous calcaneal bursa (SCB) in cattle are mainly caused by pressure sores and directly penetrating wounds. We describe the clinical, ultrasonographic and radiographic findings and outcomes in cattle diagnosed with this condition, including postoperative complications and postoperative survival times. Medical records of 29 cattle with a mean age of 4.1 years were reviewed. Twelve animals (41.4%) showed septic inflammation of the SCB only (group 1) and 17 cattle (58.6%) had a concurrent bone infection at the calcaneal tuber (group 2). Eleven cattle (37.9%) were euthanized after diagnosis due to poor prognosis. Eighteen (62.1%) patients underwent surgical treatment and 15 cattle attained full recovery with a median cumulative postoperative survival time of 23.0 months. Surgically treated patients of group 1 had a success rate of 100%, compared with 70% in group 2. Group 2 cattle with septic inflammation of the SCB and concurrent bone infection had more postoperative complications and tended to have shorter postoperative survival times. In conclusion, cattle exclusively suffering from septic SCB and treated by surgery had a good prognosis.

**Abstract:**

Septic subtendinous calcaneal bursitis in cattle commonly results from hock lesions, and less commonly from penetrating wounds. The goal of this retrospective study was to describe clinical and diagnostic imaging findings, outcomes, postoperative complications and postoperative survival times (SURV-T) in cattle with this condition. Clinical data from 29 cattle with a mean age of 4.1 years were included. Twelve (41.4%) cattle were assigned to group 1 (septic bursitis only) and 17 (58.6%) to group 2 (septic bursitis, concurrent bone infection at the calcaneal tuber (CT) and lesions of the superficial digital flexor tendon. Eleven cattle (37.9%) with comorbidities were euthanized after diagnosis due to poor prognosis. Surgical treatment was performed in 18 (62.1%) patients of which 15 showed full recovery and a median cumulative SURV-T of 23.0 months. The success rate of surgically treated patients was 100% (8/8) in group 1 and 70% (7/10) in group 2. There was no statistically significant association (*p* > 0.05) between the duration of septic bursitis and concurrent bone infection at the CT with occurrence of postoperative complications and SURV-T. However, there was a clear trend favoring more postoperative complications and shorter SURV-T in cattle with concurrent CT bone infection. In conclusion, cattle with septic subtendinous calcaneal bursitis exclusively have a good prognosis, provided adequate surgical treatment is performed.

## 1. Introduction 

Integument lesions, such as circumscribed hairless areas, scabs, decubital wounds and inflammatory swellings at exposed, peri-articular bony protuberances, are commonly described in cubicle-housed, free-stall [1,2,3] and tie-stall dairy cows [4,5,6]. Such skin lesions on the legs of cows commonly occur at the lateral tarsal region and the lateral and medial aspects of the calcaneal tuber (CT). They can substantially negatively impact animal welfare [5,7,8]. Causes of these pressure sores include management- and housing-associated factors [2,6,9]. However, hock lesions in cattle may also result from direct trauma following cuts or lacerating wounds, which are presented to the veterinarian in most cases after some delay as infected wounds associated with moderate-to-severe swelling [10,11,12]. However, in contrast to the horse [13], direct, penetrating wounds implicating tarsal synovial cavities (bursae, joint, tendon sheaths) occur less frequently in bovines [10,14].

In a cross-sectional study of 105 Austrian dairy farms, the mean farm prevalence of hock lesions was 50%, ranging widely from 0% to 100%. Interestingly, in 29% of these cows, their skin lesions were located at the CT region [1]. Hock lesions occur predominantely on the lateral tarsal aspect and more rarely on the medial aspect of the CT [1]. From these originally superficial skin lesions, septic inflammation of the subtendineous calcaneal bursa (SCB) may develop by a secondary pathway of infection [10,12,14]. 

Three synovial bursae can be differentiated anatomically in the CT region. The SCB is located between the Achilles tendon, the CT and the superficial digital flexor tendon (SDFT), permitting this tendon to glide smoothly over the cartilage layer covering the plantar aspect of the CT. The smal *Bursa tendinis calcanei* is located between the Achilles tendon (gastrocnemius tendons) and the distal end of the accessory tendon and the CT. Additionally, there is a subcutaneous calcaneal bursa located between the skin and the ligamentous cap over the CT formed by the SDFT [15,16]. The lumen of the SCB extends approximately 9 cm proximally and 7 cm distally from the CT. The SCB communicates 100% with the *Bursa tendinis calcanei* and, in addition, in 39% of cases with the subcutaneous calcaneal bursa [17].

Septic inflammation of the SCB may subsequently result in infection and osteolysis of the CT, the Achilles tendon and the SDFT, located within the bursa, and also rarely in septic arthritis of the tarsocrural joint [10]. On clinical examination, a painful and mild-to-severe swelling can be observed over the complete proximodistal extent of the SCB in the CT area, associated with moderate-to-severe lameness. In addition, if decubital or traumatic penetrating wounds are present, continuous or discontinuous serous, serofibrinous or purulent discharge from the infected bursa might be observed [10,12]. 

Tentative diagnosis of septic inflammation of the SCB can be made by a thorough orthopedic examination of an existing wound and swelling around the CT area, followed by careful probing of the wound to explore its depth and the direction of any fistulous tract [12,18,19]. For a definitive diagnosis in such cases, ultrasonographic and radiographic examinations are highly recommended [13,19,20]. The ultrasonographic examination protocol and the characteristic sonopathological findings of septic bursits of the SCB in cattle have already been described [12,21,22]. 

In contrast to the horse [13], no evidence-based studies have been carried out until now with regard to the prognosis and therapeutic success after surgical intervention in a suitably large number of cattle suffering from infection of the SCB, with the exception of a case report describing outcomes in two cows [12].

The aim of this retrospective study was to evaluate the clinical findings, diagnostic approaches and outcomes in cattle affected by septic SCB. In cattle that underwent surgical treatment, we aimed to illustrate the surgical procedure, associated postoperative complications and outcomes, including improvements in locomotion and postoperative survival times (SURV-T).

## 2. Materials and Methods

### 2.1. Patients

We retrospectively analyzed patient records of cattle with septic inflammation of the SCB that were treated at the University Clinic for Ruminants of the University of Veterinary Medicine in Vienna from January 2001 to December 2018. Animals exhibiting aseptic inflammation of the subtendineous and/or subcutaneous calcaneal bursa were excluded. 

Breed, age, sex, pregnancy status, days in lactation and locomotion score (LS) at admission and discharge, duration of bursitis before admission, topical clinical findings, ultrasonographic and radiographic findings, diagnoses, surgical and medical treatment and duration of hospitalization were recorded and evaluated. Reasons for non-treatment after diagnosis, prevalence and type of postoperative complications and final outcome after surgical treatment were assessed. The cumulative SURV-T values of successfully treated cattle were evaluated in September 2020 by telephone interviews with farmers and by accessing the Cattle-Database of AgrarMarkt Austria (AMA).

### 2.2. Clinical Examination and Diagnostic Imaging 

After obtaining a complete history of each patient, clinical and orthopedic examinations, including locomotion scoring [23], were performed. The hair of the affected tarsal region was clipped, and the area cleaned. Patients were then restrained on a tilt table in lateral recumbency allowing for better and safer examination of lesions/wounds and swellings of the CT region. All clinical findings were recorded. A sterile probe was carefully inserted into the bursa via a fistulous tract to explore its extent and determine visually if any discharge was coming from the bursa. Functional hoof trimming of all claws was also performed to check for additional lesions.

Affected regions were investigated in all patients ultrasonographically, using a 5–10 MHz linear probe, either in the standing animal or in lateral recumbency. A standardized protocol was followed that included imaging of the Achilles tendon with its insertion, the SDFT over its course on the CT, the bursal cavity, the bone contour of the complete CT surface, the cranially adjoining plantarolateral and plantaromedial pouches of the tarsocrual joint and the tendon sheath of the caudal tibial and lateral digital flexor tendon plantaromedially. The entire region of interest was scanned in proximodistal and lateromedial planes, both transversally and longitudinally [12,19,22]. The echogenicity of the inflammatory exudate and the presence or absence of flow phenomena in the infected bursa were used to determine the quality of effusion. In cases where the type of inflammatory effusion (serous, serofibrinous, fibrinous or purulent) could not be determined ultrasonographically, the affected bursa cavity was punctured using a needle with 1.6 mm lumen diameter (14 gauge), and the yielded sample was examined macroscopically. 

Radiographic examinations of the CT region were not generally performed in patients with infection of the SCB. However, it was performed in all cases with ultrasonographic evidence of CT bone involvement. Radiographic views of the CT region were taken preferably in one or two slightly different dorsolateral–plantaromedial oblique directions, in proximodistal and lateromedial directions [12,13].

The final diagnosis was made after combining clinical, orthopedic, ultrasonographic, radiographic and other pertinent findings. After consideration of the general condition, age and pregnancy status of the patients, a prognosis was presented to owners to facilitate their decision for treatment or euthanasia of the animal.

All cattle evaluated retrospectively were hospitalized at the University Clinic for Ruminants at the University of Veterinary Medicine in Vienna from January 2001 to December 2018, whereby standardized biosecurity measures had to be fulfilled. The cattle population in Austria has been officially free from bovine herpesvirus infections, responsible for IBR/IPV/IBP, since 1999. Further, only cattle originating from BVD/MD- (BVD virus: pestivirus) free herds or after presenting a negative antibody test for single animals can be referred to this clinic by local vets. All cattle were also tested for BVDV and BVDV antibodies on arrival at the clinic. 

During hospitalization, all patients were monitored for systemic infections twice daily by measuring their rectal temperatures and assessing their pulse and respiratory rates, feed intakes and defecation habits.

### 2.3. Surgical Treatment Procedure 

#### 2.3.1. Patient Preparation

Prior to thorough examination, each animal was restrained in lateral recumbency, using a hydraulic surgical tilt table with the lateral aspect of the affected hindlimb facing uppermost for the surgical approach. Patients suffering from infection of the SCB had their hindlimbs restrained with a strap positioned at the mid-level of the metatarsus, so that there was free access to the injured area in the CT region for any manipulation. In all patients the tarsal region, beginning from the distal third of the tibia down to the proximal third of the metatarsus, was clipped. This area was then cleaned diligently. 

All cattle that underwent surgery were sedated using xylazine (0.05 mg/kg i.v., Sedaxylan® 20 mg/mL—Injektionslösung, Eurovet Animal Health BV, Bladel, NL), and they received regional intravenous anesthesia. An elastic tourniquet was applied approximately 15 cm proximal of the CT, using two bandage rolls to compress adequately the medial and lateral saphenous veins [12]. Local anesthetic (procaine hydrochloride, Procamidor^®^, 20mg/mL, 400 mg, 40 ml, Richter Pharma AG, Wels, Austria) was injected into the lateral plantar metatarsal vein approximately at the level of the tarsometatarsal joint. Finally, the wound and the complete plantar tarsal region, including the proximo-distal extent of the affected bursa area, were prepared antiseptically and disinfected.

#### 2.3.2. Surgical Treatment

A detailed description of the surgical treatment method for septic inflammation of the SCB with and without concurrent bone infection of the CT and with lesions of the SDFT in two bovines has been published recently [12]. Surgical treatment included extensive wound debridement with removal of excessive granulation tissue, creation of access to the bursa via the penetrating wound tract, additional creation of two drainage portals at the proximolateral and the proximomedial pouch of the bursa and one drainage portal at the level of the most distal extent of the bursa at the laterodistal aspect (each with a diameter of approximately 15 mm), followed by meticulous removal of all exudate (serofibrinous, fibrinous, purulent) from the bursal cavity, using a curette (from each portal), and lavage of the wound and bursa. This treatment permitted good visualization of the glide surface of the CT via a wide bursal access. In cases of additional bone infection at the CT and involvement of the SDFT (laceration, partial or complete rupture and infection), meticulous curettage of the necrotic bone at the CT was performed and either injured and infected parts of the SDFT were removed, or the proximal and distal ends of the ruptured and infected SDFT were transected and removed at a level exhibiting a vital appearance [12]. 

After debridement of the wound, the bursal cavity and other infected tissues, the bursa was lavaged meticulously with 2–3 L of sterile 0.9% saline solution (Physiologische Kochsalzlösung 0.9%, Fresenius Kabi, Graz, Austria) containing 0.1% povidone–iodine (Vet-Sept® Lösung 10%, aniMedica GmbH, Senden-Bösensell, Germany). The surgical wounds were drained, using an infusion tube (Intrafix^®^ Primeline intravenous administration set, B. Braun Melsungen AG, Melsungen, Germany), which was inserted through two surgical openings and then fastened with knots. Polyurethane-soft-foam (Ligasano® polyurethane-soft-foam, Ligamed medical products GmbH, Cadolzburg, Germany) was inserted as drainage material into the large wound cavity [12,24]. Subsequently, 5 ml of ampicillin (Ampicillin “Vana” 200 mg/mL^®^—Injektionssuspension für Tiere, Vana GmbH, Vienna, Austria) was instilled into the bursal cavity. 

All wounds were covered with polyurethane-soft-foam dressing and a thick, padded compression bandage was applied over the tarsal region. For patients treated for septic bursitis only, the bandage was completed by application of an elastic adhesive dressing in at least two layers (Optiplaste^®^ längselastische Klebebinde, 10.0 × 2.5 m^2^ roll, BSN medical Medizinprodukte GmbH, Vienna, Austria). In all patients in which infected bone at the CT and/or parts of the SDFT had been removed, the highly flexible tarsal joint with the wound was temporarily immobilized by application of a Robert Jones bandage or a fiberglass cast using four cast rolls (Cellacast^®^ Longuette 12 cm, Lohmann & Rauscher, Vienna, Austria). The fiberglass cast extended from the distal third of the tibia to the mid-metatarsus. The tarsal joint was fixed by the cast at the physiological 150° angle without cast fenestration. The limb distal to the fiberglass cast was covered with a bedded bandage to avoid distal venous and lymphatic congestion [12]. 

#### 2.3.3. Peri- and postoperative medical and topical treatments

All patients were housed postoperatively in either deep-bedded tie or loose stalls. Peri- and postoperative antibiotic treatments comprised procaine penicillin and dihydrostreptomycin (Peni-Strepto® 200/200mg/mL, 8 mg/kg i.m., Virbac Laboratoires, Carros, France), ampicillin (Ampicillin ‘Vana’^®^ 200 mg/mL, 10 mg/kg i.m., Vana GmbH, Vienna, Austria), oxytetracycline (Engemycin^®^ 100 mg/mL, 10 mg/kg i.m., Intervet GesmbH, Vienna, Austria), cefquinome (Cobactan^®^ 2.5%, 1 mg/kg i.m., Intervet GesmbH, Vienna, Austria) or ceftiofur (Eficur^®^ 50 mg/mL, 1 mg/kg s.c., Laboratorios Hipra S.A., Girona, Spain) for approximately three to ten days, depending upon bone infection and group allocation. Antimicrobial choice depended upon availability during the 18-year recording time of the study. 

The nonsteroidal, anti-inflammatory treatment generally comprised ketoprofen (Rifen® 100 mg/ml, 3 mg/kg i.m./i.v., Richter Pharma AG, Wels, Austria) for three days. 

Bandages were changed for the first time two days after surgery, with the animal again restrained in lateral recumbency after sedation with xylazine. The fiberglass cast was bisected in its lateral and medial lengths using an oscillating saw, and both the front and rear halves of the cast, the cotton-padded bandage and the polyurethane-soft foam drainage were removed. Following local anesthesia, the surgical wound was again flushed with sterile 0.9% saline solution containing 0.1% povidone–iodine. A new polyurethane-soft foam drainage and a padded bandage were applied, as performed on the day of surgery. In patients that received additional immobilization, the bisected fiberglass cast was finally re-applied using 5 cm-wide tape (Tesa^®^ Gewebeband 4541, Vienna, Austria) to brace the cast-halves together, or a Robert Jones bandage was applied. The wound was lavaged in the same way, always after sedation and application of local anesthesia, twice or three times weekly, with an interval of two to four days, using the same solutions as mentioned above during the bandage changes for the subsequent three weeks [12]. 

When the wounds were covered and completely sealed with vital granulation tissue, as was the case typically within 3–4 weeks after surgery, and when the initial lameness had distinctly decreased to a mean LS of approximately 2/5 (mild lameness), the patients were discharged. The local veterinarian was advised to remove the bandage, the fiberglass cast or the Robert Jones bandage after the patient had returned to the farm. Additionally, patients were housed on the farm separately in a well-padded recovery pen. The wound was cleaned daily by the farmer using a water hose, and a wound ointment or wound spray was applied topically. This treatment was repeated until the wound size had decreased further and epithelialization had progressed considerably, and lameness had more or less completely disappeared such that the animal could return back to the herd.

### 2.4. Statistical Analysis

Statistical analysis was performed using Microsoft Excel (Excel 2019®, Microsoft Corp., Redmond, WA, USA) and SPSS^®^ Statistics for Windows, version 25.0 (IBM Corp., Armonk, NY, USA). Mean, standard deviation, median, minimum and maximum values were calculated. Patients were divided into two groups depending on bursal and bone involvement: group 1 exclusively included patients with septic bursitis; and group 2 patients had septic bursitis, concurrent osteolysis of the CT and infection of the SDFT (in three cases).

The non-parametric Chi-squared test was used for examination of relationships between categorical variables, such as between concurrent infection of the CT in patients with septic bursitis and the occurrence of postoperative complications, and between the duration of septic bursitis before admission and the occurence of postoperative complications. 

The Mann–Whitney test was used to test for relationships between LS at the time of treatment and the occurrence of CT bone infection, and between LS at the time of treatment and occurrence of postoperative complications. 

The Spearman rank correlation coefficient was computed to test for possible associations between patient age, SURV-T and LS at the time of treatment and LS at time of discharge from the clinic. Using SPSS^®^ Statistics, the SURV-T of group 1 and group 2 patients and those patients with postoperative complications were assessed and were graphically presented using the Kaplan–Meier survival plot. Statistical significance was defined as *p* < 0.05 against a two-sided null hypothesis of no difference.

## 3. Results

### 3.1. Patients

Medical records of a total of 29 cattle with septic bursitis of the SCB were reviewed. Most cattle were Fleckvieh (*n* = 22; 75.9%) or Holstein Friesian (*n* = 6; 20.7%); one was a Limousin cow. 

At the time of admission to the clinic, the mean age of these 29 cattle was 4.1 years (±1.6; median: 3.8; min: 1.8; max: 7.5). Twenty-six were female (89.7%) and three (10.3%) were male. Of the females, 25 were in lactation (96.2%), and one was a pregnant heifer. Four of the 25 cows (16.0%) were pregnant, 15 (60.0%) were non-pregnant and the reproduction status of six cows was unknown. Fourteen cows (56.0%) were in the first trimester of lactation, four cows (16.0%) in the second, one cow in the last trimester and in six cows (24.0%) the days in milk were unknown. 

Anamnestic data showed that pretreatment had been carried out by the referring veterinarians in 16 (55.2%) patients by systemic administration of antibiotics in 13 patients, by administration of NSAIDs in eight patients, by both medical treatments in seven patients, and by topical medication in three patients. For the other thirteen patients, no information about pretreatment was available.

### 3.2. Clinical Findings, Diagnoses and Localization

All cattle were examined clinically and orthopedically. At the time of admission, the 29 cattle exhibited a mean LS of 3.5 (±1; median: 3; min: 1; max: 5). A secondary pathway of infection was identified as causative for septic bursitis in 24 patients (82.8%), resulting from originally superficial skin lesions and decubital sores at the CT area. A direct penetrating wound at this location was identified in five patients (17.2%). 

**Figure 1:**
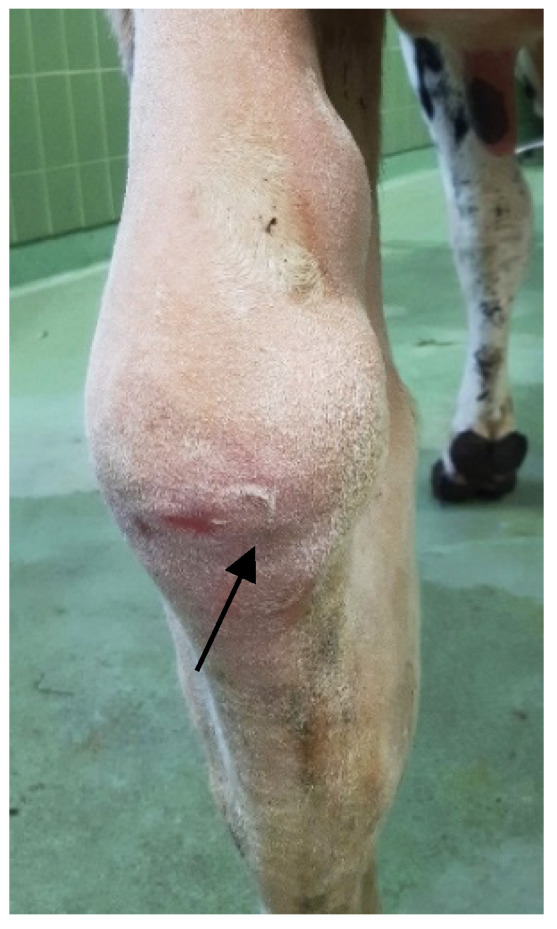
Caudomedial view of the left calcaneal region of a 6.5-year-old Holstein cow (115 days in lactation, non-pregnant) showing evident swelling over the calcaneal tuber (CT) region and proximally thereof, indicating severe effusion of the subcutaneous calcaneal bursa (SCB) caused by a wound on the plantar aspect (black arrow) of four-weeks duration.

At the time of admission, the duration of septic bursitis was already longer than eight days in 26 cattle (89.7%), whereas in three other patients (10.3%) the bursal infection had a history of three to five days (caused by a direct penetrating injury). Infection of the SCB was located in 13 patients (44.8%) in the right hindlimb, in tweve (41.4%) in the left hindlimb and four (13.8%) patients suffered from bilateral septic bursitis. Therefore, out of these 29 cattle, 33 cases of septic subtendinous calcaneal bursitis were diagnosed (Figure 1).

Rectal temperature on the day of admission was a mean of 38.7 °C (±0.7) in all 29 patients (median: 38.5 °C, min: 38.0 °C; max: 41.0 °C). At admission, the mean rectal temperature in the eighteen treated cattle was 38.5°C (±0.3) (median: 38.4° C; min: 38.0 °C; max: 39.3 °C), and it was 39.1 °C (±0.9) (median: 39.0 °C; min: 38.0 °C; max: 41.0 °C) in eleven cattle showing comorbidities necessitating euthanasia. In total, eleven cattle had elevated rectal temperatures (>38.8 °C); six of these belonged to the group with comorbidities necessitating euthanasia, and the other five had elevated temperatures (38.9–39.3 °C) for only the first two or three days.

**Figure 2:**
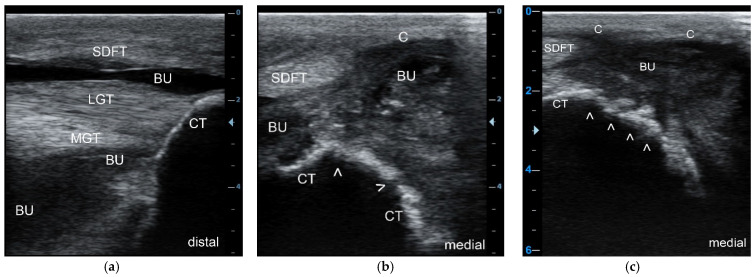
Longitudinal sonogram (5 MHz linear) of the CT region at the insertion site of the Achilles tendon (**a**), and transverse sonograms (5 MHz linear) of the plantaromedial (**b**) and medial aspect (**c**) of the proximal CT showing a septic purulent bursal effusion (**a**) and bone involvement (infection) in this cow; the bursal cavity (BU) is highly enlarged due to the presence of heterogeneous hypoechoic exudate with an anechoic, partly hypoechoic to echoic and “snow-flurry-like” appearance, the latter in particular at the medial aspect of the bursal cavity with flow phenomena (**b**,**c**), indicative of a purulent exudate. Due to the severe effusion, the superficial digital flexor tendon (SDFT) is displaced plantarily, as is the bursal capsule (C). The contour of the CT appears normal at the insertion site of the lateral (LGT) and medial gastrocnemius tendons (MGT) (**a**), but highly irregular (white arrows) at the plantaromedial (**b**) and, in particular, at the medial aspect of the CT (**c**) indicating extensive osteolysis.

Ultrasonographic examination was conducted in all 29 patients and the following was identified: an inflammatory, heterogenous effusion in all 33 cases of bursitis (100%), gas pockets within the bursal cavity in 15 cases (45.5%), bone lesions at the CT in 16 cases (48.5%) (Figure 2a–c), and lesions of the SDFT with loss of echogenicity and loss of parallel fiber arrangement leading to the diagnosis of partial and total tendon necrosis/rupture in three cases (9.1%), respectively. All three cattle with SDFT lesions had concurrent bone osteolysis of CT.

**Figure 3:**
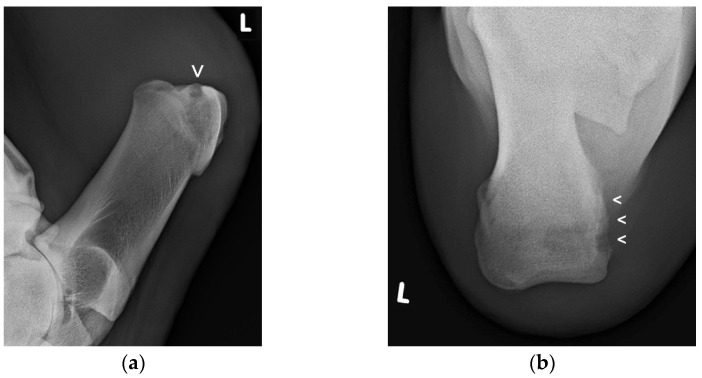
Lateromedial (**a**) and proximodistal radiographic views (**b**) of the left CT region of this cow showing an extended radiolucent zone (small arrow/s) at the proximomedial aspect of the CT, indicating bone osteolysis (Figure 3a,b courtesy of the Clinical Unit of Diagnostic Imaging, Vetmeduni Vienna, Austria).

Radiographic examination (Figure 3a,b) was performed in 19 patients (65.5%): in fifteen cattle on the day of admission, in three cattle later on during hospitalization, and in one patient, radiographs were made at admission and again later. The various radiographic findings are shown in Table 1. 

The quality of the inflammatory exudate characteristic of septic bursitis could be revealed by macroscopic evaluation of spontaneous discharges from the wound tracts of 16 cases (48.8%), or by ultrasonographic examination (in all), by diagnostic puncture, or intra-operatively in 15 cases (45.5%). Diagnostic puncture of the affected bursa was performed in ten cases (30.3%). Based on the character of the synovial effusion in all 33 cases of septic bursitis, we could distinguish septic serous (*n* = 3; 9.1%), septic serofibrinous (*n* = 3; 9.1%), fibrinous (*n* = 2; 6.1%), fibrinopurulent (*n* = 7; 21.2%) and purulent exudates (*n* = 18; 54.5%). Bacteriological culture from eight samples with purulent exudate revealed mixed infections with *Trueperella pyogenes*, *E. coli*, *Proteus spp.,* and *beta-hemolytic streptococci.*

Following final diagnosis, cattle were divided into two groups: those to be euthanized and those to undergo treatment. Cattle to be treated were further divided into groups 1 and 2, depending upon bursal and CT bone involvement: twelve animals (41.4%) were assigned to group 1 (septic SCB only) and 17 (58.6%) to group 2 (septic SCB and concurrent bone infection at the CT, and in three cattle infection and partial or complete rupture of the SDFT) (Table 2). 

### 3.3. Reasons for Non-Treatment

Eleven cattle (37.9%) were euthanized due to poor prognosis after diagnosis and discussion of the findings with the owners (Table 2). These patients had severe comorbidities, such as painful claw disorders (*n* = 6), acute laminitis (*n* = 2), purulent endometritis (*n* = 3), septic arthritis of the tarsal joint (*n* = 2), valvular endocarditis (*n* = 1), severe abdominal diseases (*n* = 3) and multiple, severe decubital wounds at other limb locations (stifle, coxal tuber, ischial tuber, precarpal; *n* = 6).

### 3.4. Outcome, Postsurgical Complications, and Hospitalization Time

In total, 18 cattle (62.1%) with septic bursitis of the SCB received surgical treatment, with eight patients from group 1 (44.4%) and ten from group 2 (55.6%); one of the latter had complete rupture of the SDFT. Successful outcomes were achieved for fifteen patients (Table 2).

Postoperative complications occurred in seven of 18 patients (38.9%) within three to 17 days after first surgical intervention (Table 2). These included new purulent discharge from the bursa (*n* = 7), new osteolysis at the CT (*n* = 5), small sequestrum formation at the CT (*n* = 2) and pathological fracture of the CT (*n* = 1). Of these seven patients, four (57.1%) were treated successfully by repeated wound debridement, bone curettage, sequestrotomy, repeated lavage of the bursa and the wound and local and systemic antibiotic treatment. Three of these four successfully treated cattle with postoperative complications belonged to group 2. The remaining three patients (42.9%), all from group 2, were euthanazed due to extensive infection of the CT and a pathological fracture of the CT, resulting in poor prognosis.

The eleven animals without postoperative complications were treated systemically with antibiotics for a mean of 4.6 days (±2.5; median: 4.5; min: 1; max: 9) and with NSAIDs for a mean of 3.0 (±1.1; median: 3.0; min: 1; max: 4) days. The four patients with postoperative complications, but successful outcomes, were treated systemically with antibiotics for a mean of 12.0 days (±7.0; median: 11.5; min: 4; max: 21) and with NSAIDs for a mean of 5.3 (±2.6; median: 4.5; min: 3; max: 9) days.

The eleven patients without postoperative complications were hospitalized for a mean for 16.1 days (± 7.0; median: 18.0; min: 4.0; max: 25.0), whereas the four cattle with postoperative complications, but successful outcomes, had a mean hospitalization time of 28.5 (± 18.4; median: 21.5; min: 16.0; max: 55.0) days (Table 3). The LS of the 29 cattle at admission was a mean of 3.0 (±1.0; median: 3.0; min: 2; max: 5), whereas at discharge the 15 successfully treated cattle had a mean LS of 1.8 (±0.6; median: 2.6; min: 2; max: 3) (Table 3).

In total, 29 cattle were presented to the clinic with septic bursitis of the SCB during the 18-year observation period. Eleven of these patients (37.9%) were euthanized after diagnosis, and 18 (62.1%) received surgical treatment. Eleven (out of 18) (61.1%) patients showed full recovery without postoperative complications. Seven patients (38.9%) developed postoperative complications, four of which could be successfully treated (57.1%). Fifteen out of 18 (83.3%) surgically treated cattle had a good final outcome, comprising all eight patients of group 1 (100%) and seven patients of group 2 (70.0%) (Table 2).

### 3.5. Postoperative Survival Time (SURV-T)

Two patients were still alive at the time of this retrospective analysis, with ages of 5.2 and 5.3 years, and survival times of 29.0 and 31.0 months, respectively. The mean cumulative SURV-T of all 15 cattle with successful outcomes was 20.5 (±14.9; median: 23.0; min: 6.0; max: 43.3) months. Comparison of cumulative SURV-T of patients between the two groups revealed a mean SURV-T of 24.1 (±5.1; median: 23) months for group 1 cattle and a mean SURV-T of 13.4 (±5.6; median: 3.1) months for group 2 cattle. Patients without postoperative complications showed a mean SURV-T of 21.3 (±4.5; median: 23.0) months, whereas the four patients with complications had a mean SURV-T of 13.1 (±7.4; median: 10.7) months. Two patients were culled because of problems related to lameness, one cow six months after discharging due to development of a sole ulcer in the contralateral limb. Another cow with a SURV-T of 8.3 months developed a pelvic fracture due to an accident, but she exhibited a normal gait until this incident. The longest survival time of 43.3 months was determined in a group 2 cow with concurrent infection of the CT.

### 3.6. Statistical Results

The Spearman rank correlation coefficient test revealed a significantly (*p* = 0.03) negative correlation (r = −0.502) between the ages of cattle at admission and their SURV-T; clearly, the younger the cattle at the time of surgery, the greater their SURV-T. 

LS at admission and at discharge against SURV-T, respectively, revealed a very low correlation (r = −0.116 and r = 0.282) without statistical significance (*p* = 0.64 and *p* = 0.30). However, the LS score at admission showed high statistical significance (*p* = 0.006) and good correlation (r = 0.676) with LS at discharge, implying that the lower the initial LS was at the time of admission, the lower it was at the time of discharge after treatment. Neither CT infection (*p* = 0.089), nor involvement of the SDFT (*p* = 0.163), nor duration of bursal infection (*p* = 0.582) at the time of admission showed a statistically significant association with LS at admission. Moreover, even LS at admission was not statistically significantly associated (*p* = 0.548) with the occurrence of postoperative complications.

**Figure 4:**
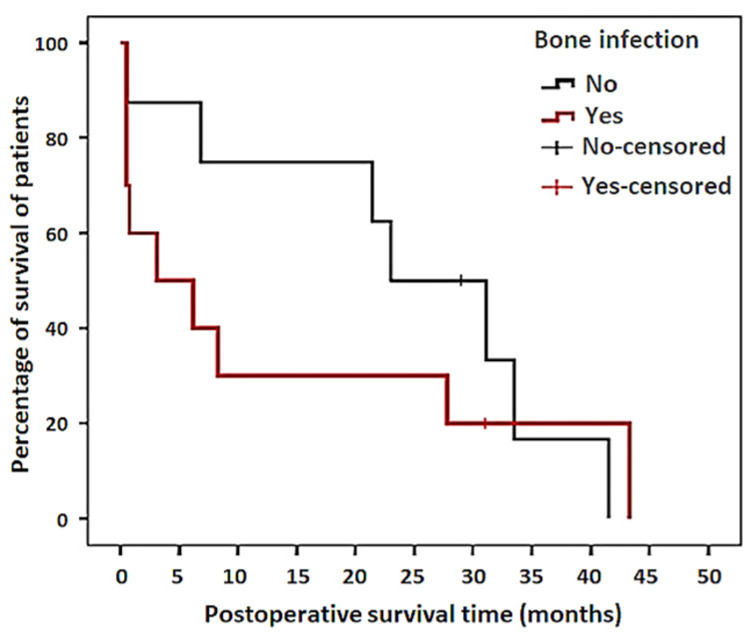
Kaplan–Meier survival curve comparing the cumulative postoperative survival of patients of group 1 (septic bursitis only—No) with patients of group 2 (concurrent bone infection—Yes).

No statistically significant association (*p* = 0.420) could be determined between the presence of septic bursitis with concurrent CT bone infection and SURV-T. However, these group 2 patients showed a clear trend toward lower SURV-T in the Kaplan–Meier survival analyis compared to patients with only septic bursitis (Figure 4). 

**Figure 5:**
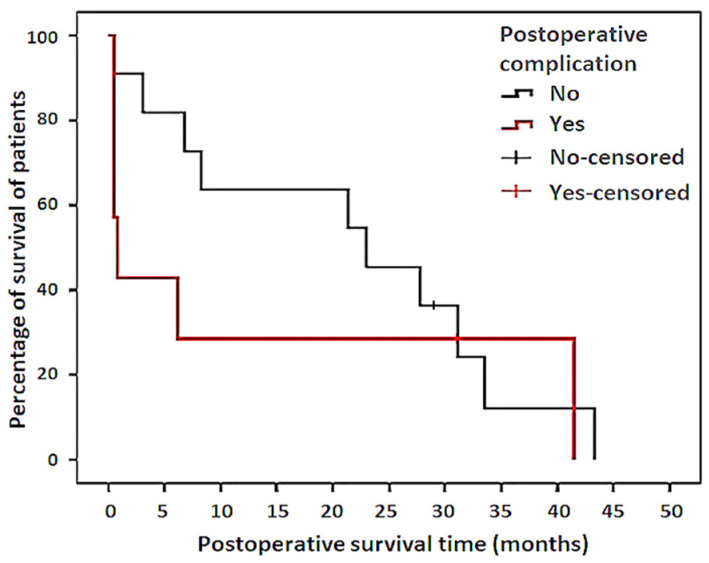
Kaplan–Meier survival curve comparing the cumulative postoperative survival of patients without (No) and with postoperative complications (Yes).

A statistically significant association (*p* = 0.04) was determined between the presence of septic bursitis with concurrent CT bone infection and the occurrence of postoperative complications. However, no statistically significant association (*p* = 0.357) could be determined between the occurence of postoperative complications and SURV-T. Nevertheless, in patients with postoperative complications, a clear trend toward a lower SURV-T in the Kaplan–Meier survival analysis was observed, compared to patients without complications (Figure 5). Moreover, no statistically significant association (*p* = 0.06) was determined between the duration of bursal infection (more or less than eight days) at the time of admission and the occurrence of postoperative complications.

## 4. Discussion

This study is the first retrospective evaluation of surgical treatment, postoperative outcomes and postoperative survival times in a relatively moderate sample of 29 bovines suffering from infection of the SCB. To date, only one case report has recently described the surgical treatment of septic bursitis of the SCB in two cows, and their outcomes [12]. While treatment regimens for septic SCB have been described briefly in three older textbooks, they are without evidence-based information on long-term outcomes [10,18,25]. In contrast, in 2003, a similar retrospective analysis regarding outcomes after surgical intervention in 24 horses with septic bursitis of the SCB was published [13].

In cattle, infections of the SCB and the CT most frequently originate from skin lesions and decubital sores on the lateral aspect of the CT following a secondary pathway [1,10,19]. It is relevant that both anatomic structures are located only approximately 8–10 mm under the skin surface [15,16,17]. In the present study, which included 29 cattle with septic inflammation of the SCB, it was mostly a secondary pathway of infection originating from decubital wounds in the CT region that could be affirmed in 82.8% of the patients; the other five cases were caused by directly penetrating wounds. A similar distribution of relevant causes for septic bursitis of the SCB in cattle has been reported by other authors [10,18,25]. In contrast, direct penetrating wounds involving this bursa are the most common pathway for infection in horses [13]. 

Unfortunately, cattle patients exhibiting lameness and wounds are generally presented for treatment at an advanced stage of infection [11,14,25]. In the present evaluation, infection of the SCB had a mean duration of more than eight days in 89.7% of cattle, which is quite similar to reports regarding horses [13]; bursal infection had a mean duration of three days and a maximum of five days in only three cattle.

At admission, the 29 cattle with septic bursitis showed a mean LS of 3.0 (median: 3.0) (out of 5 scores [23]), which is similar to the mean lameness score of 3.2 (median: 3.0) observed in horses with the same disorder [13]. In these cattle, the LS was neither associated with bone infection of the CT and involvement of the SDFT, the duration of septic bursitis before admission, nor with the occurence of postoperative complications. As with horses [13], LS in cattle is a poor guide for estimation of the progress of septic calcaneal bursitis. These results can be explained partially by the variability of individual sensitivity to pain in cattle [26]. Nevertheless, the LS of these cattle at admission showed a highly significant and relatively strong correlation (r = 0.676) with LS at discharge, implying that the lower the initial LS was, the lower it was at discharge after treatment.

For a reliable diagnostic evaluation of wounds associated with inflammatory swelling in the CT region accurate clinical and orthopedic examinations followed by ultrasonographic and radiographic imaging are essential, and can be concluded by puncture of the bursal cavity [12,13,19]. In particular, ultrasonographic units used for bovine reproductive health checks, equipped with 5–10 MHz linear probes, are well suited for differentiation of all soft-tissue structures of the bovine tarsal and CT region [22]. Ultrasonographic examination of the suspected region, following a standardized protocol [22], allows for early identification of bursal effusion, as well as early detection of bony alterations of the CT in respect of bone infection. Ultrasonography is particularly suitable, as it can image osteolytic lesions at early stages of development, compared to radiography [12,19,20]. Furthermore, even the SDFT and the Achilles tendon can be scanned with accuracy ultrasonographically from proximal to distal, searching for tendon fiber disruption and infection. Accurate ultrasonographic inspection of the suspected area enables a comprehensive assessment of the quantity and quality of inflammatory bursal effusion, and whether or not there is CT bone involvement, consolidating a well-founded prognosis and facilitating the decision as to the merits of subsequent radiographic scans [20,22]. To reveal the comprehensive extent of long-lasting CT bone infection, the radiographic examination must include oblique and proximodistal projections [13,20]. Moreover, it has to be kept in mind that characteristic radiographic signs of osteitis/osteomyelitis can be identified only approximately 10–14 days after the onset of bone infection, which is exactly the time period necessary for sufficient bone demineralisation [20].

In cases without visible discharge from the wound tract, the bursal cavity can be punctured to classify the quality of exudate (serous, serofibrinous, fibrinous, purulent), and from the aspirated sample a bacteriological culture and antibiotic sensitivity testing can be initiated [13,19]. In the present study, approximately half of the patients had a visible discharge from the bursal wound tract, and this was mostly purulent or fibrinopurulent.

In all 29 cattle with septic inflammation of the SCB, even an accurate physical examination was included so as not to overlook comorbidities, which may have had a negative impact on the overall prognosis of the patient. Thus, in eleven (out of 29) cattle, after confirming the diagnosis, euthanasia was decided because of multiple, severe additional disorders that diminished prognosis. As in horses with septic bursitis of the SCB, but perhaps much more relevant in cattle, it is not only the prognosis that matters, but treatment costs have to be discussed with farmers prior to any surgical intervention. As this retrospective study included patients covering a time span of 18 years, it is difficult to compare treatment costs. However, for the most recent three years, farmers have had to expect mean treatment costs of approximately EUR 720, restricted to material and medication expenses, not costs for surgery. For comparison, the mean cost of a dairy cow in Austria in 2020 purchased at auction was EUR 1850 [27]. 

Treatment prior to referral at the clinic was instituted in 55.2% of the 29 cattle and included antibiotics, NSAIDs and local wound lavage. In a retrospective study of 24 horses, 71.0% of them were treated by the referring veterinarian using the same medication as in this study—wound lavage and bandages [13]. Nonetheless, treatment of septic bursitis of the SCB by application of systemic antibiotics and NSAIDs exclusively is never a promising approach. Effective treatment of this condition should always include surgical intervention [12,13,18]. This comprises meticulous debridement of the existing wound and the tract into the bursal cavity, ensuring visual access to the cartilaginous glide surface of the CT, complete removal of inflammatory exudate from the bursal cavity, meticulous curettage of the necrotic parts of the CT and/or debridement of infected parts of the SDFT, the creation of drainage portals at the proximolateral/proximomedial pouches and at the distolateral pouch of the bursa, followed by meticulous lavage of the bursal cavity and drainage [12,18,25]. However, in contrast to horses, which require general anesthesia for surgical treatment of this disorder [13], cattle can be handled successfully after sedation, with placement in lateral recumbency on a surgical table and application of regional intravenous retrograde anesthesia [12,18]. 

An important component of treatment, besides the surgical treatment of bursal infections, is the administration of peri- and postoperative systemic antibiotics and NSAIDs. Depending on the occurrence of postoperative complications, antibiotics and NSAIDs in this study were administered for a mean of 4.6 (median: 4.5) and 3.0 (median: 3.0) days in patients without complications, and for a mean of 12.0 (median: 11.5) and 5.3 (median: 4.5) days in patients showing postoperative complications, respectively. In other words, group 1 patients treated only for septic bursitis, with the exception of one animal in group 1, required much shorter medical treatment (antibiotics and NSAIDs), compared with group 2 patients who additionally underwent bone curettage. Admittedly, peri- and postoperative pain management with NSAIDs was only for a limited period, mainly due to the current drug licensing regulations for farm animals [28]. Besides NSAIDs, patients were sedated with xylazine and received procaine for local anesthesia prior to surgery, and also for postoperative wound treatments (repeated lavage). For more multimodal pain management, epidural anesthesia using procaine 2% [28], or butorphanol [28] could have been used after redesignation of the latter for use in farm animals [29]. Again, in contrast, horses receiving comparable surgical interventions were treated with antibiotics for a mean of 32 days; however, seven out of 24 horses (29%) showed complications and required repeated surgical debridement [13]. As monogastrics, horses could receive medications orally after the first seven days, and received NSAIDs for a median of nine days [13], which is much longer than reported here for cattle.

Wounds located over the protuberance of the CT are at risk of poor healing due to tension and constant motion of the highly mobile tarsal joint. Furthermore, partial removal of infected bone tissue from the CT may debilitate bone stability and weaken the insertion site of the Achilles tendon. Therefore, strict box rest and sufficient immobilisation of the tarsus to limit motion in this area are essential aftercare aspects of treatment. Immobilization of the tarsal joint can be achieved by application of a Robert Jones bandage or a fiberglass cast during the hospitalization period [12,13]. Both types of immobilisation bandages were well tolerated in the cohort studied. 

As with a retrospective study in horses with septic bursitis of the SCB, the 29 bovine patients were divided into two groups, depending on the severity of the disease; group 1 patients (*n* = 12) included all the cattle showing septic bursitis exclusively, and group 2 patients (*n* = 17) had septic bursitis and concurrent bone infection of the CT, and rarely infection with partial or complete rupture of the SDFT. The treatment success rate in all group 1 patients was 66.7%, 100% in treated group 1 patients and 41.2% in all (70% in treated) group 2 patients that returned to their farms. In a similar study in equines, only 75% of those with wounds involving the SCB survived, and concurrent infection of the CT was associated with a guarded prognosis, where only 44% of horses survived [13]. 

Cows with concurrent bone infection of the CT had a significantely higher occurrence of postoperative complications, compared to cattle with only septic bursitis. Thus, six of seven bovine patients showing postoperative complications had an initial diagnosis of concurrent bone infection. Similar results were described in horses suffering from the same condition; horses progressing with normal healing after one single surgical intervention had a survival rate of 86%, whereas horses with postoperative complications showed a survival rate of merely 14% [13]. Postoperative complications in cattle were observed five to 17 days after surgery as revealed by ascending LS scores, renewed onset of purulent discharge and increased swelling. These complications included osteolysis, bone sequestration and pathological fracture of the CT that could not be controlled in three cases. Minor complications were probably caused by insufficient removal of inflammatory exudate from the bursa and the communicating bursa *tendinis calcanei* [13,17]. However, in other cases, it is possible that the bone infection initially involved too large an extent of the CT, which could not be clearly imaged using radiography. In particular, in the case of bone infection, even surgical removal of all visibly infected bone down to hard vital bone is sometimes very difficult. The surgeon must be satisfied at the time that all infected bone tissue is removed [12,13]. Alternatively, too much bone removal limits the function of the remainder, such as in the case of the CT, which provides stability to the insertion site of the Achilles tendon [13]. This can result in pathological fracture of the CT or a pathological avulsion fracture of the Achilles tendon insertion [10], as happened as a catastrophic complication in one of our patients.

Assuredly, postoperative complications can prolong hospitalization time. Accordingly, the eleven cattle without postoperative complications showed a mean of 16.1 days (median: 18.0), a shorter hospitalization period than the mean of 28.5 days (median: 21.5) for the four cattle with complications. Similarly distinct results were reported in horses after surgical bursal intervention with a mean hospitalization time of 20 days without versus a mean of 33 days for horses with postopertive complications [13]. However, hospitalization period as a measure of treatment success must be used with caution because it is highly dependent on owner compliance, the referring veterinarian and their ability to perform bandage changes on the farm.

In the present study, there were no statistically significant associations between the duration of septic bursitis before treatment and the occurrence of postoperative complications and SURV-T, nor between the duration of septic bursitis and the LS score of cattle at admission. Similar results were recorded in horses in which the duration of septic bursitis showed no association with postoperative survival [13]. In contrast to horses, the target of successful healing of this condition in cattle is restricted to ability to return to a normal walking gait (no faster), which improves the prognosis of cattle with only septic bursitis.

In the aggregate for all 15 cattle with successful outcomes, they had a mean age of 3.5 (median 3.0) years at the time of surgery and a mean cumulative SURV-T of 20.5 (median: 23.0) months. This corresponds to prolonged production for approximately two more lactation periods. During the period of 2005 to 2018 the mean milk price for Austrian farmers ranged between EUR 0.2948 and 0.3696 per kg [30]. The mean milk loss for cows suffering from septic subtendinous calcaneal bursitis has never been reported. However, milk losses for Austrian dairy cows showing locomotion scores ≥ 4 have been calculated with means of 319 and 602 kg of milk, depending upon the breed [31]. Alternatively, the mean annual milk yield of an Austrian dairy cow ranges from 7625 kg (for Brown–Swiss cows) and 7834 kg (for Fleckvieh cows) to 9117 kg (for Holstein cows) [32]. However, for these 15 cattle, their mean life expectancy was approximately eight months shorter than the 6.3 years [32] for an average Austrian dairy cow. 

In the present study, most cases of septic subtendinous calcaneal bursitis were caused by ascending infections originating from initially superficial pressure sores. Therefore, prevention must focus on avoidance of deep sepsis at the CT region, particularly by early and adequate treatment of decubital wounds, by debridement, application of a bandage and temporary separation of affected cattle into well-padded recovery boxes. Nevertheless, prevention of these decubital wounds should start by optimizing the quality of cubicles and barns and their bedding and by regular hoof trimming of dairy cows approximately three times a year for prevention of painful claw lesions [6,14,31].

## 5. Conclusions

Effective treatment of septic inflammation of the SCB should always include surgical wound debridement, creation of additional portals, removal of exudate and infected tissue from the bursal cavity, followed by lavage and drainage. According to the results of this retrospective study, cattle showing septic bursitis of the SCB exclusively had an excellent final outcome and favourable prognosis. A trend could be observed whereby cattle with concurrent bone infections of the CT tended to develop more postoperative complications, which were associated with poorer outcomes. These results underline the importance of a definitive diagnosis at an early stage of septic bursitis, and the initiation of early, effective surgical treatment, even in cattle. 

## Figures and Tables

**Table 1:** Radiographic findings in 19 cattle with septic bursitis of the SCB.

Findings	Number of Observations	%
Soft tissue swelling	19	100.0
Osteolysis/osteomyelitis of CT	12	63.2
Gas pockets within bursal cavity	7	36.8
Periosteal bone proliferation	4	21.1
Sequestrum formation at CT	3	15.8
Bone fragments at CT	2	10.5
Normal bone contour of CT	3	15.8

SCB: subtendinous calcaneal bursitis; CT: calcaneal tuber.

**Table 2:** Overview of 29 cattle with septic bursitis of the SCB divided into group 1 (septic SCB only) and group 2 (septic SCB with concurrent bone and SDFT involvement), number of euthanized cattle, number of treated cattle, number of cattle with postoperative complications and cattle with successful outcomes; SCB—subtendinous calcaneal bursitis; SDFT—superficial digital flexor tendon.

Overview of Treated and Non-Treated Cattle	Group 1		Group 2		Total	
	*n*	%	*n*	%	*n*	%
Number of cattle at admission	12	41.4	17	58.6	29	100
Number of euthanized cattle after diagnosis	4	36.4	7	63.6	11	37.9
Number of treated cattle	8	44.4	10	55.6	18	62.1
Number of cattle with postoperative complications	1	12.5	6	60	7	38.9
Number of successfully treated cattle with complications	1	100	3	50	4	57.1
Number of cattle euthanized after initial treatment	0	0	3	50	3	42.9
*Total number of euthanized cattle*	*4*	*33.3*	*10*	*58.8*	*14*	*37.9*
*Total number of successfully treated cattle*	*8*	*100*	*7*	*70*	*15*	*83.3*

**Table 3:** Overview of the age of 29 cattle at admission, their locomotion score at admission and at discharge after successful treatment, and hospitalization times for cattle with and without postoperative complications; SCB—subtendinous calcaneal bursitis; LS—locomotion score; SD—standard deviation.

	mean	SD	median	min	max
Age of cattle with septic bursitis of the SCB at admission in years; *n* = 29	4.1	1.6	3.8	1.8	7.5
Age of successfully treated cattle at admission in years; *n* = 15	3.5	1.3	3.0	1.8	6.0
LS (1 5) of all cattle at admission; *n* = 29	3.0	1.0	3.0	2.0	5.0
LS (1 5) of successfully treated cattle at admission; *n* = 15	2.9	1.2	3.0	2.0	5.0
LS (1 5) of successfully treated cattle at discharge; *n* = 15	1.8	0.6	2.6	2.0	3.0
Duration of hospitalization for cattle without postoperative complications in days; *n* = 11	16.1	7.0	18.0	4.0	25.0
Duration of hospitalization for cattle with postoperative complications in days; *n* = 4	28.5	18.4	21.5	16.0	55.0

min—minimum; max—maximum.
